# Quantitative Analysis of Somatostatin and Dopamine Receptors Gene Expression Levels in Non-functioning Pituitary Tumors and Association with Clinical and Molecular Aggressiveness Features

**DOI:** 10.3390/jcm9093052

**Published:** 2020-09-22

**Authors:** Álvaro Flores-Martinez, Eva Venegas-Moreno, Elena Dios, Pablo Remón-Ruiz, Noelia Gros-Herguido, M. Carmen Vázquez-Borrego, Ainara Madrazo-Atutxa, Miguel A. Japón, Ariel Kaen, Eugenio Cárdenas-Valdepeñas, Florinda Roldán, Justo P. Castaño, Raúl M. Luque, David A. Cano, Alfonso Soto-Moreno

**Affiliations:** 1 Unidad de Gestión de Endocrinología y Nutrición. Instituto de Biomedicina de Sevilla (IBiS), Hospital Universitario Virgen del Rocío/CSIC/Universidad de Sevilla, 41013 Sevilla, Spain; alvflomar@gmail.com (Á.F.-M.); evam.venegas.sspa@juntadeandalucia.es (E.V.-M.); elenadiosfuentes@gmail.com (E.D.); pjremonruiz@gmail.com (P.R.-R.); ngros.h@gmail.com (N.G.-H.); amadrazoatutxa@gmail.com (A.M.-A.); 2 Maimonides Institute for Biomedical Research of Córdoba (IMIBIC), 14004 Córdoba, Spain; marvazbor@gmail.com (M.C.V.-B.); justo@uco.es (J.P.C.); bc2luhur@uco.es (R.M.L.); 3 Department of Cell Biology, Physiology and Immunology, University of Córdoba, 14004 Córdoba, Spain; 4 Hospital Universitario Reina Sofía, 14004 Córdoba, Spain; 5 CIBER Fisiopatología de la Obesidad y Nutrición (CIBERObn), 14004 Córdoba, Spain; 6 Department of Pathology, Hospital Universitario Virgen del Rocío, Instituto de Biomedicina de Sevilla (IBIS), Hospital Universitario Virgen del Rocío/CSIC/Universidad de Sevilla, 41013 Sevilla, Spain; mangel.japon.sspa@juntadeandalucia.es; 7 Servicio de Neurocirugía, Hospital Universitario Virgen del Rocío, 41013 Sevilla, Spain; kaenariel@hotmail.com (A.K.); eugeniocarde@hotmail.com (E.C.-V.); 8 Servicio de Radiología, Hospital Universitario Virgen del Rocío, 41013 Sevilla, Spain; florivict@gmail.com

**Keywords:** pituitary tumor, invasion, somatostatin receptor, dopamine receptor, non-functioning pituitary tumors

## Abstract

The primary treatment for non-functioning pituitary tumors (NFPTs) is surgery, but it is often unsuccessful. Previous studies have reported that NFPTs express receptors for somatostatin (SST_1-5_) and dopamine (DRDs) providing a rationale for the use of dopamine agonists and somatostatin analogues. Here, we systematically assessed SST_1-5_ and DRDs expression by real-time quantitative PCR (RT-qPCR) in a large group of patients with NFPTs (*n* = 113) and analyzed their potential association with clinical and molecular aggressiveness features. SST_1-5_ expression was also evaluated by immunohistochemistry. SST_3_ was the predominant SST subtype detected, followed by SST_2_, SST_5_, and SST_1_. DRD2 was the dominant DRD subtype, followed by DRD4, DRD5, and DRD1. A substantial proportion of NFPTs displayed marked expression of SST_2_ and SST_5_. No major association between SST_s_ and DRDs expression and clinical and molecular aggressiveness features was observed in NFPTs.

## 1. Introduction

Non-functioning pituitary tumors (NFPTs) are characterized by the absence of clinical symptoms related to pituitary hormone overproduction. NFPTs usually present with symptoms related to mass effects such as visual impairment, headaches and hypopituitarism [1]. Histologically, NFPTs comprise a heterogeneous group of tumors, consisting of gonadotropin-storing tumors, silent pituitary tumors and null cell adenomas. NFPTs are usually benign but a substantial proportion of these pituitary tumors show an aggressive behavior with local invasion, and increased risk of regrowth or recurrence after surgery [2,3]. However, the underlying mechanisms for this aggressive behavior are largely unknown [4,5]. The primary line of treatment for these tumors is transsphenoidal resection but it is often surgically incomplete and/or clinically unsuccessful [6]. Currently, there is no medical therapy approved for the treatment of NFPTs [7,8]. NFPTs express dopamine receptors (DRDs) and several studies have described the use of dopamine agonists for the treatment of NFPT patients, albeit with variable success [7,8,9]. NFPTs also express somatostatin receptors (SST_1-5_) thus providing a theoretical rationale for the pharmacological treatment with somatostatin analogues [10]. However, in the limited number of studies published to date, SSTs expression has been analyzed at the mRNA level [11,12,13] or by immunohistochemistry (IHC) [14,15] but rarely both methods have been applied simultaneously in the same study. This is an important point, as a consistent method for evaluating SST_1-5_ expression in pituitary tumors has yet to be implemented in a clinical pathology setting.

Several immunohistochemical biomarkers have been studied for their potential association to aggressive features in NFPTs such as the Ki-67 proliferative index, p53 and ERα (recently reviewed in [16]). However, more studies are needed to firmly establish their utility in clinical practice.

The aim of this study was to thoroughly analyze DRDs and SSTs expression in a cohort of well-characterized NFPT samples by both real-time quantitative PCR (RT-qPCR) and immunohistochemistry (IHC). In addition, we aim to determine the potential association of DRDs and SSTs expression with relevant clinical and molecular aggressive features of NFPTs. 

## 2. Experimental Section

### 2.1. Patients and Samples

This study was conducted following the ethical standards of the Helsinki Declaration of the World Medical Association and approved by the IBiS-Virgen del Rocio Hospital Ethics Committee (0208-N-17) and written informed consent was obtained from all patients. The biobank of the public health system of Andalusia, Spain (Seville Node) coordinated the collection, processing, management and assignment of the biological samples used in this study, according to the standard procedures established for this purpose. This retrospective descriptive study includes patients diagnosed with NFPTs who underwent transsphenoidal surgery for newly or recurrent pituitary tumors by the same team of neurosurgeons at the Virgen del Rocío University Hospital (Seville, Spain) between 1998 and 2017. Patients were evaluated by a multidisciplinary team composed of neuroradiologists, neurosurgeons, and endocrinologists. The initial diagnosis of NFPT was established based on the absence of biochemical hormonal overproduction and lack of associated clinical symptoms. After surgery, the diagnosis was verified histologically and immunohistochemically for pituitary hormones (Growth hormone, prolactin, adrenocorticotropic hormone, luteinizing hormone, follicle-stimulating hormone and thyroid-stimulating hormone) by an experienced pathologist. One hundred and thirteen patients whose archival tissues were available were included in this study. Clinical variables were collected to analyze potential associations between these variables and DRDs and SSTs expression. Specifically, cavernous sinus invasion and tumor size data were acquired from magnetic resonance images. Cavernous sinus invasion was evaluated using the Knosp classification. Knosp grade 3 and 4 were considered invasive. Surgical cure was defined as absence of tumor on MRI at 3 months after surgery. Tumor regrowth was defined as evidence of regrowth of tumor remnant on MRI within 2 years of follow-up.

### 2.2. Histopathology and Immunohistochemistry

Formalin-fixed paraffin-embedded tissues from NFPTs were obtained and four tissue microarrays (TMAs) were constructed. Cores were taken from areas of the paraffin block recognized as tumor tissue by evaluation of hematoxylin and eosin-stained sections by an expert pathologist. Duplicates of each NFPTs and four cores of normal pituitary tissue (obtained from autopsies) were included in each TMA. SSTs and E-cadherin immunohistochemistry as well as the used score system has been previously described [17,18]. DRD expression was not evaluated since, to the best of our knowledge, no commercial reliable antibodies are available. The following SSTs antibodies were used: SST_2_ (Abcam, Cambridge, UK, ab134152) 1:100; SST_3_ (Abcam, Cambridge, UK, ab137026) 1:750; SST_5_ (Abcam, Cambridge, UK, ab109495) 1:100. Immunohistochemical analysis for E-cadherin (ready-to-use, clone 36, VENTANA, Roche, Basel, Switzerland, catalog number 790-4497), Ki-67 (clone 30-9, VENTANA, Roche, Basel, Switzerland, catalog number 790-4286), and p53 (clone DO-7, VENTANA, Roche, Basel, Switzerland, catalog number 790-2912) were performed using an automated immunostainer system (VENTANA, Roche, Basel, Switzerland) following the manufacturer’s specifications. Ki-67 index was determined as the percentage of tumor cells with nuclei positive for Ki-67 in relation to total cells in at least 3 different spots.

### 2.3. RNA Isolation, Reverse Transcription, and Analysis of Gene Expression by Quantitative Real-Time PCR

Somatostatin receptor (SST_1_-SST_5_) and dopamine receptor (DRD1-DRD5) expression by real-time quantitative PCR (RT-qPCR) was quantified using primers previously reported [19]. SST_4_ and DRD3 were not analyzed since their expression is negligible in NFPTs [10,11,20]. The expression values of target genes were normalized to ACTB mRNA levels, as in previous studies from our group [21,22]. Technical details on RNA extraction, reverse-transcription and qPCR quantification have been reported elsewhere [18,23].

### 2.4. Statistical Analysis

Normality of the data was assessed using the Kolmogorov–Smirnov test. The categorical variables are described as percentages and frequencies. Non-normally distributed data are shown as median values with interquartile ranges (IQR). For normally distributed mean ± SD are shown. ANOVA and Student’s t test were used for parametric variables and Kruskal–Wallis and Mann–Whitney test for nonparametric variables. Chi-square was used for categorical variables. Spearman’s rank correlation coefficient was used for correlation analysis between continuous variables. P values were adjusted for multiple comparisons by the Benjamini–Hochberg false discovery rate method. Statistical analysis was performed using SPSS software version 25.0 for Windows (SPSS, Chicago, IL, USA). *p* values < 0.05 were considered statistically significant.

## 3. Results

### 3.1. Patient and Sample Characteristics

A total of 113 NFPTs tumors from patients were analyzed. The clinical characteristics of the study population are shown in Table 1. All of the patients with NFPT had macroadenomas. Fifty-two tumors (46%) were invasive. Only four tumors had high Ki-67 levels (>3%). Thirty-two tumors (28.3%) did not show any hormonal expression, as assessed by immunohistochemistry (Table 2). Surgery achieved complete resection in 41 patients (36.3%). Tumor regrowth within two years of follow-up was observed in 15% of the patients (17 out of 58) with tumor remnant after surgery.

### 3.2. Receptor Expression Levels in NFPTs

**Figure 1:**
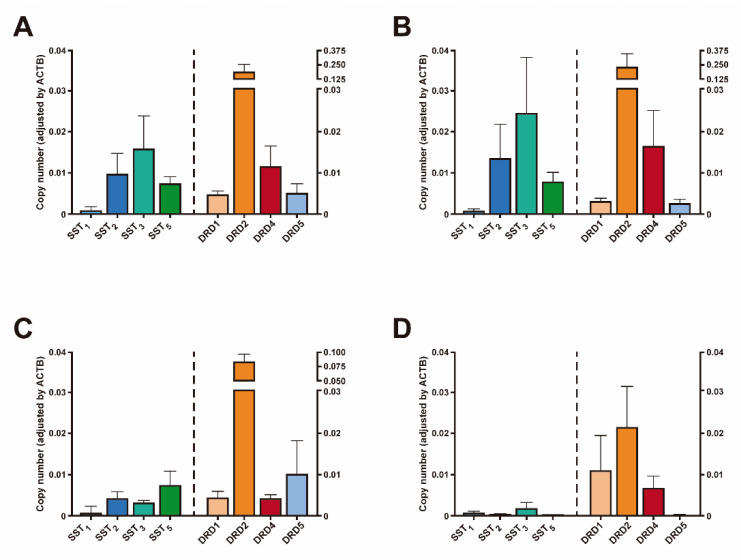
Somatostatin receptors (SSTs) and dopamine receptors (DRDs) expression in NFPTs. Expression profile of SSTs and DRDs in: (**A**) NFPTs, gonadotropin-storing adenomas, null cell adenomas and silent corticotroph adenomas; (**B**) only in gonadotropin-storing adenomas; (**C**) only in null cell adenomas; and, (**D**) only in silent corticotroph adenomas. mRNA expression levels were measured by quantitative RT-PCR. Copy numbers of each transcript was adjusted by the expression levels of a control gene (ACTB). Data are shown as mean ± SEM.

Mean mRNA expression levels of SSTs and DRDs from NFPTs are shown in Figure 1A. SST_3_ was the predominant SST subtype detected, followed by SST_2_, SST_5_, and SST_1_. DRD2 was the dominant DRD subtype, followed by DRD4, DRD5, and DRD1 (Figure 1A). Expression levels for the major histopathological types of NFPTs (i.e. Gonadotropin-storing tumors; (*n* = 67), null cell tumors (*n* = 32) and silent corticotroph tumors (*n* = 10)) are also shown in Figure 1 (only two plurihormonal and two silent Growth Hormone-storing tumors were found in our cohort).

Gonadotropin-storing tumors show an expression pattern similar to what it is observed in the whole NFPT population, with SST_3_ and DRD2 displaying the highest expression levels (Figure 1B). Null cell adenomas (defined as tumors lacking any immunohistochemical hormone expression), however, display a different SSTs and DRDs expression pattern, with SST_5_ being the predominant SST subtype expressed, followed by SST_2_, SST_3_ and SST_1_, while DRD2 was the dominant DRD subtype, followed by DRD5, DRD1 and DRD4 (Figure 1C). Silent corticotroph tumors (SCTs) are defined as pituitary tumors with immunohistochemical expression of ACTH but without biochemical hypercortisolism and no clinical symptoms of Cushing’s disease. SCTs are considered aggressive pituitary tumors [24]. In SCTs, SST expression was, in general, low. SST_3_ showed the highest expression, but levels were notably lower compared to gonadotropin-storing tumors and null cell tumors. DRD expression pattern in SCTs was also different compared to the other two major histological subtypes, being DRD2 the predominant DRD subtype followed by DRD1, and DRD4. Notably, DRD5 expression levels were extremely low (Figure 1D).

In NFPTs, expression levels of SST_2_ were directly correlated to SST_1_ (*r* = 0.34, Spearman FDR adjusted *p* = 0.015), SST_3_ (*r* = 0.31, *p* = 0.008) and SST_5_ (*r* = 0.35, *p* = 0.015). SST_1_ expression levels were directly correlated to SST_5_ (*r* = 0.35, *p* = 0.015). Expression levels of DRD1 were directly correlated with DRD5 expression levels (*r* = 0.33, *p* = 0.018). In addition, several significant correlations were found between SSTs and DRDs. Specifically, SST_1_ expression levels were directly correlated to DRD1 (*r* = 0.35, *p* = 0.015) and DRD5 expression levels (*r* = 0.33, *p* = 0.018). SST_2_ expression levels were directly correlated to DRD5 expression levels (*r* = 0.43, *p* = 0.001). Expression levels of SST_3_ were directly correlated to those of DRD2 (*r* = 0.36, *p* = 0.001). SST_5_ expression levels were directly correlated to DRD4 (*r* = 0.29, *p* = 0.04) and DRD5 expression levels (*r* = 0.38, *p* = 0.008).

### 3.3. Association between Clinical Characteristics of NFPTs and SSTs or DRDs mRNA Levels

We evaluated whether there was an association between SSTs or DRDs mRNA expression levels and key clinical features of aggressiveness in NFPTs. However, no significant differences were found between invasion, surgical cure or tumor regrowth and expression levels of any SST and DRD receptor. Additionally, no significant correlations between SSTs or DRDs expression and tumor size, age or sex was observed. We performed the same analysis in two histological groups of NFPTs: gonadotropin-storing tumors and null cell adenomas (the number of SCTs in our series was too low to make meaningful analyses). Specifically, no significant differences were found between invasion, surgical cure or tumor regrowth and expression levels of any SST and DRD receptor in gonadotropin-storing tumors and null cell adenomas. However, in gonadotropin-storing tumors we observed a negative correlation between DRD2 expression and tumor size (*r* = −0.36, *p* = 0.03), while in null cell adenomas, a positive correlation was found between DRD5 mRNA expression and tumor size (*r* = 0.66, *p* = 0.004). Of note, no difference in the gene expression level of any SSTs and DRDs was found between gonadotropin-storing tumors and null cell adenomas.

### 3.4. SSTs Expression in NFPTs as Assessed by Immunohistochemistry 

**Figure 2:**
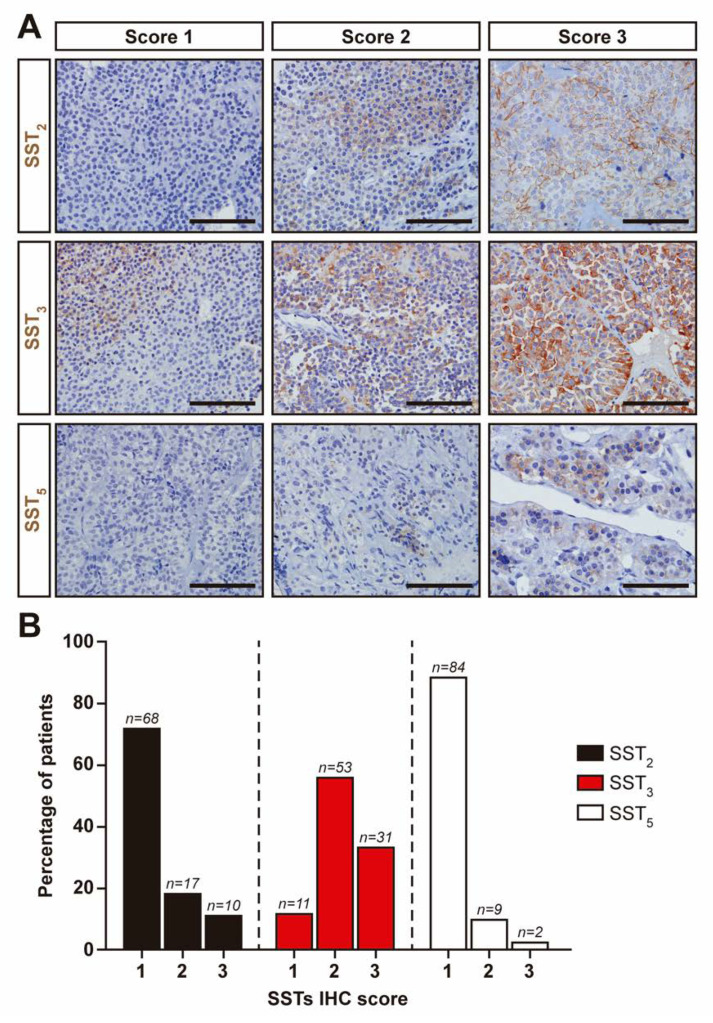
Immunohistochemical detection of somatostatin receptors (SSTs) in NFPTs assessed by immunohistochemistry. (**A**) Representative pictures of SST_2_, SST_3_ and SST_5_ immunohistochemical scores in NFPTs. Score 1, no or only cytoplasmic immunoreactivity; score 2, membranous immunoreactivity in less than 50% of cells; score 3, membranous immunoreactivity in more than 50% of cells. Scale bar: 50 μm. (**B**) Percentage of NFPTs for immunohistochemistry (IHC) scores (SST_2_, SST_3_ and SST_5_).

Evaluation of SSTs expression by IHC, while perhaps less sensitive, is very useful in the clinical setting. We decided to evaluate SSTs expression with commercial antibodies that have been previously used in several studies, including from our own group. Of the 113 NFPTs included in the study, we could analyze SSTs expression by IHC in 95. We were unable to obtain reliable immunoreactivity with the SST_1_ antibody (Abcam, ab137083) in any NFPT sample and thus, IHC scoring was not performed for SST_1_. Representative images of SSTs in normal pituitary and the different scores in NFPTs are shown in Figure 2.

Most of the tumors expressed SST_3_ (88%, Figure 2B). In contrast, the number of tumors with substantial expression of SST_2_ and SST_5_ was low (28 and 11%, respectively). Gonadotropin-storing tumors and null cell tumors display a similar IHC score pattern, with a large number of tumors showing high SST_3_ scores but low SST_2_ and SST_5_ scores (Figure 2B). Surprisingly, no differences in SST_2_, SST_3_ and SST_5_ mRNA expression levels among their respective different IHC scores was found (*p* = 0.06, 0.16 and 0.07, respectively). Similar to what we observed when SSTs expression were examined by RT-qPCR, no differences were found for any SST between age, sex, tumor size, invasion, surgical cure or tumor regrowth and IHC scores. 

### 3.5. Evaluation of Molecular Markers Associated with Aggressive Features in NFPTs

We sought to investigate the association of proliferation indexes, namely Ki-67 and p53, with aggressive features in our series of NFPTs. No association was found between Ki-67 levels and age, tumor size, invasion, surgical cure or tumor regrowth. Additionally, we did not find increased Ki-67 levels in giant (tumor size larger than 40 mm) NFPTs. However, we need to note that most of NFPTs showed low Ki-67 levels with only four tumors displaying high Ki-67 levels (>3%). Similar results were obtained with p53 immunohistochemistry (i.e., only four tumors also showed high p53 levels). Three of these tumors also displayed high Ki-67 levels and exhibited recurrence.

**Figure 3:**
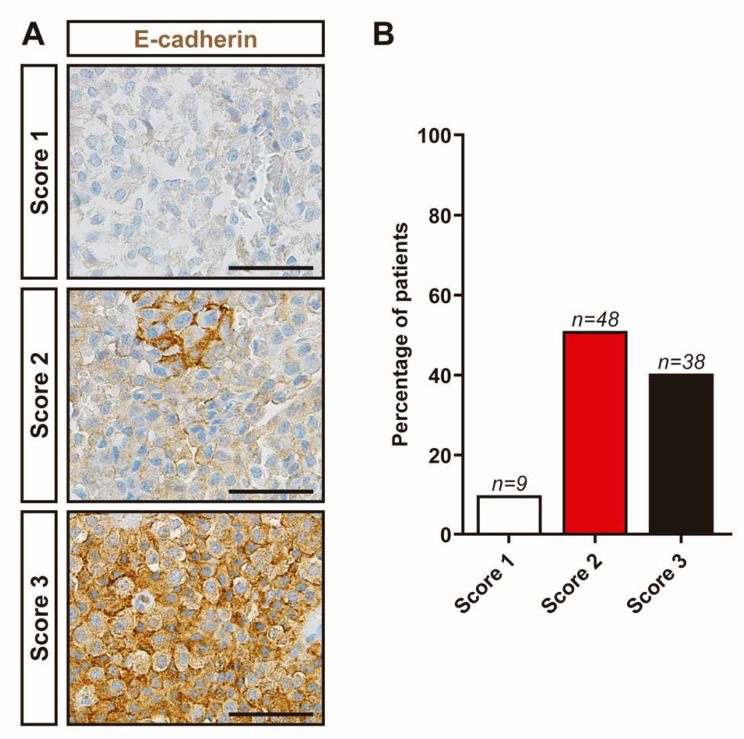
Accumulation of E-cadherin in NFPTs assessed by immunohistochemistry. (**A**) Representative pictures of E-cadherin immunohistochemical scores in NFPTs. Score 1, no or very low immunoreactivity; score 2, membranous immunoreactivity in less than 50% of cells; score 3, membranous immunoreactivity in more than 50% of cells. Scale bar: 100 μm. (**B**) Percentage of NFPTs for IHC scores. Score 1: 9.47%; score 2: 50.52% and score 3: 40%.

Next, we evaluated E-cadherin expression levels in NFPTs. Representative images of E-cadherin IHC semiquantitative scores in NFPTs are shown in Figure 3. 

Nine tumors displayed no or extremely low membranous staining (Figure 3B). Forty-eight tumors displayed moderate membranous immunoreactivity (less than 50% of the tumor cells), while 38 tumors exhibited strong membranous immunoreactivity in more than 50% of the cells. However, we did not find statistically significant differences in tumor size, invasiveness and tumor regrowth among the three different E-cadherin IHC scores. Additionally, no differences in E-cadherin IHC score between gonadotropin-storing and null cell tumors were observed. Finally, no significant correlations between SSTs or DRDs expression and Ki-67, p53 index level or E-cadherin IHC score were observed.

## 4. Discussion

Here, we determined the expression profile of SSTs and DRDs in a large cohort of patients with NFPTs by RT-qPCR. SST_3_ was the predominant SST subtype expressed, followed by SST_2_, SST_5_ and SST_1_. Regarding DRDs expression, DRD2 was the predominant DRD subtype, followed by DRD4, DRD5, and DRD1. Our present findings compare favorably with previous studies using similar methodological approaches [11,13,20,25,26]. Our cohort of patients with NFPTs was composed of different histological subtypes, in similar proportions to what has been observed in previous large series of this type of tumors [27]. Thus, most of NFPTs were gonadotropin-storing tumors, followed by null cell tumors and SCTs. Only two plurihormonal and two silent GH-producing tumors were found in our cohort, confirming the rarity of these histological subtypes. The number of null cell tumors in our study may have been overestimated in our study as immunohistochemical expression of pituitary-specific transcription factors was not available. Nevertheless, the SSTs and DRDs expression pattern was similar between gonadotropin-storing and null cell tumors. In contrast, SCTs exhibited a distinct pattern of expression of SSTs and DRDs compared with the two other major histological subtypes. Thus, SST_3_ was the predominant SST subtype in SCTs, but levels were markedly lower compared to both gonadotropin-storing tumors and null cell adenomas. Similarly, even though DRD2 was the predominant DRD subtype in SCTs, expression levels were clearly lower than those found in both gonadotropin-storing tumors and null cell adenomas. In line with our data, two previous studies evaluating DRDs expression by RT-qPCR also reported lower DRD2 expression levels in SCTs compared to ACTH-negative pituitary adenomas [13,28]. The SSTs and DRDs expression patterns in SCTs are similar to those found in ACTH-secreting pituitary adenoma, as expected, since both tumor types share the same pituitary corticotroph lineage origin.

Dopamine agonists (DA) have been considered as a potential medical therapy for recurrent NFPTs [7,8]. Preclinical studies largely support this notion. Thus, DA inhibit gonadotropin secretion and cell growth of NFPTs in vitro [29,30]. The antitumor activity seems to involve both direct and indirect mechanisms such as antiangiogenic effects [29]. However, there is not enough solid evidence to recommend DA in routine clinical practice in this setting [7]. It has been suggested that response to DA treatment in NFPTs may be associated to DRD2 expression [31,32]. Based on this notion, our results would suggest that SCTs are less amenable for DA treatment. Nevertheless, it is important to note that a recent study could not find a relationship between the response to DA treatment and DRD2 levels in NFPTs [9] and thus, further studies are warranted to ascertain the efficacy of DA treatment for NFPTs and its relation to DRDs presence.

Our RT-qPCR results confirm previous studies that SST_3_ is, by far, the predominant SST expressed in NFPTs [11,12,14,26]. Since the correlation between SSTs mRNA and protein expression in pituitary adenomas is not always concordant [18,33], we used IHC with commercial and widely validated antibodies to evaluate SSTs expression in NFPTs. Indeed, we found no correlation between in SST_2_, SST_3_ and SST_5_ mRNA expression levels and their respective IHC scores in NFPTs. This apparent discrepancy might be due to post-transcriptional regulation of SSTs protein synthesis among other biological processes. Differences inherent to each methodology could also account for this lack of correlation. In our case, SSTs scoring by IHC in NFPTs samples was carefully evaluated by a pathologist to ensure that only tumor tissue was analyzed. Nonetheless, IHC confirmed that SST_3_ was the SST most frequently expressed in NFPTs (84%) followed by SST_2_ and SST_5_. These results are very similar to a recent study evaluating SSTs expression by IHC [14]. In contrast, another study reported that SST_2_ was the most prevalent SST in NFPTs [15]. The discrepancies between this study and ours (and the study by Lee et al. [14]) may be due to differences in scoring criteria. 

Preclinical studies have provided evidence that somatostatin analogues have antiproliferative effects in vitro in pituitary tumors, including NFPTs [34,35]. The antiproliferative activity of SSTs appears to be dependent on its receptor selectivity and thus, they might have different effects depending on the tumor subtype. In this regard, our results show that 84% of NFPTs displayed moderate or high SST_3_ expression. SST_3_ has been long considered a potential therapeutic target for medical treatment of NFPTs. Indeed, in a very recent collaborative study, we described that SST_3_ agonists inhibit cell growth in primary cell cultures from human NFPTs as well as in a preclinical mouse model [11]. Of note, it was found that the response to SST_3_ agonist treatment was associated to SST_3_ expression levels [11]. It would be very interesting to determine whether a similar correlation exists between SST_3_ expression and clinical response to SST_3_ agonists in patients with NFPTs. The clinical development of safe SST_3_-specific agonists is thus eagerly awaited to test whether this is an effective treatment for patients with NFPTs. An additional and attractive approach is combined therapy with DA and somatostatin analogues [7].

We found that a substantial proportion of NFPTs expressed a high degree of SST_2_ and SST_5_ expression (28% for SST_2_ and 11% for SST_5_). While the proportion might be relatively low, the finding that some NFPTs produce substantial amounts of SST_2_ and SST_5_ may have potential therapeutic implications concerning the use of somatostatin analogs targeting these specific SSTs. In this regard, specific antagonists for SST_2_ have been shown to inhibit cell proliferation in vitro, in primary cell cultures of NFPTs [34]. However, a SST_5_ selective agonist actually increased NFPT cell viability [22]. Our results raise the question as to whether SST_2_ and SST_5_ agonists would be more effective in NFPTs expressing high levels of these SSTs. Proper studies evaluating different somatostatin analogues in NFPTs with different SSTs expression patterns would be very informative. 

Our results demonstrate a wide heterogeneity in SSTs and DRDs expression in NFPTs, even within the different histological subtypes. There are likely multiple reasons underlying this variability and these are not well understood. However, the emerging picture of NFPTs (and PiTNETs in general) is that they are more complex at the molecular level than previously thought and that the current classification, based mainly on histological criteria, may be somewhat limited [36,37]. In this regard, a very recent study performed an unbiased, integrated pangenomic analyses in PiTNETs and found new tumor subtypes not previously characterized [38]. Indeed, the authors analyzed SST_2_, SST_5_ and DRD2 expression and observed substantial differences in expression levels among the newly identified PiTNETs subtypes [37]. Thus, the variability in SSTs and DRDs expression in NFPTs may reflect (at least, partially) the heterogeneous genetic and molecular landscape of these types of tumors.

In an attempt to evaluate potential histological markers of tumor behavior we studied in our large group of NFPTs several immunohistochemical markers that have been previously described to be associated with aggressive features of pituitary tumors. Thus, loss of membranous E-cadherin immunoreactivity has been reported to be associated with invasion in pituitary tumors in several studies, including our own [17,39,40,41,42,43]. Nevertheless, it is important to note that most of these studies were performed in functioning pituitary tumors. Actually, in the case of NFPTs, we found no association between E-cadherin accumulation and invasion, results that are in agreement with two previous studies [44,45]. Another study found lower E-cadherin levels in invasive NFPTs [41]. However, a different antibody and scoring IHC system was used in this study, thus making it difficult to compare the results. 

The use of Ki-67 and p53 as prognostic markers of pituitary tumor behavior is controversial, with reports providing discordant results (recently reviewed in [3]). In particular, different cut-off values for Ki-67 levels have been proposed as predictors of pituitary tumor recurrence. Indeed, due to this variability, the 2017 WHO classification of pituitary tumors no longer defines a specific cut-off for Ki-67 levels. Nevertheless, the European Society of Endocrinology guidelines recommend Ki-67 index evaluation and p53 immunohistochemical evaluation when the Ki-67 index is higher than 3% but the interpretation of these results should be always considered within the clinical context of the individual patient [46]. In our study, the vast majority of NFPTs displayed low Ki-67 and p53 levels (less than 3% of the cells) in agreement with previous reports [47,48,49,50]. Only four tumors out of 95 (4.2%) showed proliferation levels higher than 3% (for each marker). This low number precludes attaining statistically significant results; however, we should note that all the NFPTs with high Ki-67 levels exhibited tumor progression. Interestingly, three of these tumors showed elevated p53 levels while only one tumor with low Ki-67 levels (out of 91) had high p53 expression levels. 

In summary, our RT-qPCR and IHC analysis of a large number of NFPTs corroborates that SST_3_ and DRD2 are the predominant receptors in these tumors, although, no associations were found between SSTs and DRDs expression and any relevant clinical and molecular aggressiveness features of NFPTs. However, our data also revealed that a considerable proportion of NFPTs displayed appreciable levels of SST_2_ and SST_5_ expression, a finding with potential therapeutic implications for the use of SST_2_ and SST_5_ agonists for NFPTs treatment. Furthermore, a combined SSTs and DRDs expression signature may provide a rationale for the potential use of multimodal therapies, at least, in specific cases.

## Figures and Tables

**Table 1:** Baseline characteristics of the study populations. IQR: interquartile ranges.

Characteristics	
Sex (number and % female)	40 (35.4)
Age at diagnosis (years, median, IQR)	58 (44–70.5)
Maximum tumor diameter at diagnosis (mm, median, IQR)	29.5 (22–39.5)
Invasiveness (% Knosp grade ≥ 3)	52 (46)
Ki-67 index (%, median, IQR)	0.54 (0.30–1.05)

**Table 2:** Non-functioning pituitary tumors (NFPTs) histological subtypes.

Histological Subtype	Number (%)
Gonadotroph-storing tumor	67 (59.3)
Null cell tumor	32 (28.3)
Silent corticotroph tumor	10 (8.8)
Plurihormonal tumor	2 (1.8)
Silent somatotroph tumor	2 (1.8)
